# Analysis of the effect of permeant solutes on the hydraulic resistance of the plasma membrane in cells of *Chara corallina*

**DOI:** 10.1007/s00709-024-02000-6

**Published:** 2024-10-23

**Authors:** Masashi Tazawa, Randy Wayne, Maki Katsuhara

**Affiliations:** 1 Yoshida Biological Laboratory, 11-1 Takehanasotoda-Cho, Yamashina-Ku, Kyoto 607-8081 Japan; 2 Laboratory of Natural Philosophy, Plant Biology Section, Cornell University, Ithaca, NY USA; 3 Institute of Plant Science and Resources (IPSR), Okayama University, 2-20-1, Chuo, Kurashiki 710-0046 Japan

**Keywords:** *Chara corallina*, Effective osmotic pressure, Hydraulic resistance, Plasma membrane, Reflection coefficient

## Abstract

In the cells of *Chara corallina*, permeant monohydric alcohols including methanol, ethanol and 1-propanol increased the hydraulic resistance of the membrane (*L*p_m_^−1^). We found that the relative value of the hydraulic resistance (^r^*L*p_m_^−1^) was linearly dependent on the concentration (*C*_s_) of the alcohol. The relationship is expressed in the equation: ^r^*L*p_m_^−1^ = ρ_m_*C*_s_ + 1, where ρ_m_ is the hydraulic resistance modifier coefficient of the membrane. Ye et al. (2004) showed that membrane-permeant glycol ethers also increased *L*p^−1^. We used their data to estimate *L*p_m_^−1^ and ^r^*L*p_m_^−1^. The values of ^r^*L*p_m_^−1^ fit the above relation we found for alcohols. When we plotted the ρ_m_ values of all the permeant alcohols and glycol ethers against their molecular weights (MW), we obtained a linear curve with a slope of 0.014 M^−1^/MW and with a correlation coefficient of 0.99. We analyzed the influence of the permeant solutes on the relative hydraulic resistance of the membrane (^r^*Lp*_m_^−1^) as a function of the external (π_0_) and internal (π_i_) osmotic pressures. The analysis showed that the hydraulic resistance modifier coefficients (ρ_m_) were linearly related to the MW of the permeant solutes with a slope of 0.012 M^−1^/MW and with a correlation coefficient of 0.84. The linear relationship between the effects of permeating solutes on the hydraulic resistance modifier coefficient (ρ_m_) and the MW can be explained in terms of the effect of the effective osmotic pressure on the hydraulic conductivity of water channels. The result of the analysis suggests that the osmotic pressure and not the size of the permeant solute as proposed by (Ye et al., J Exp Bot 55:449–461, 2004) is the decisive factor in a solute’s influence on hydraulic conductivity. Thus, characean water channels (aquaporins) respond to permeant solutes with essentially the same mechanism as to impermeant solutes.

**Supplementary Information:**

The online version contains supplementary material available at 10.1007/s00709-024-02000-6.

## Introduction

The hydraulic conductivity of plant cells (*L*p) has been studied intensively in characean internodal cells by means of the transcellular osmosis method (Kamiya and Tazawa 1956,) or by the pressure probe method (Steudle and Zimmermann 1974). In the transcellular osmosis method, an internodal cell is partitioned in two halves. Transcellular osmosis is induced by applying an osmolyte solution to one half of the cell, while the other half is in water. Water moves from the water side to the osmolyte side through the cell. Dainty and Ginzburg (1964a) found that in cells of *Chara australis*, the *L*p on the exosmosis side tended to decrease with an increase in the concentration of the sucrose solution. They proposed that this occurred because the plasma membrane in contact with the osmotic solution was dehydrated, and became compact and less permeable to water. Combining transcellular osmosis with cell ligation, Kamiya and Kuroda (1956) prepared from an internodal cell of *Nitella flexilis* two shorter cells, one having a higher osmotic pressure the other having a lower osmotic pressure than the normal one. Tazawa and Kamiya (1966) measured the *L*p of the twin cells and found that the *L*p of the cell half with a higher osmotic pressure was lower than that of the normal cell and the *L*p of the half cell with a lower osmotic pressure was higher than that of the normal cell. The dependence of *L*p on the internal osmotic pressure is consistent with the proposal of Dainty and Ginzburg (1964a).

Kiyosawa and Tazawa (1972) further studied the effects of extracellular and intracellular osmotic pressures on the hydraulic conductivity (*L*p) in cells of *Nitella flexilis* using mannitol as the external osmolyte. The intracellular osmotic pressure was modified by the transcellular osmosis/cell ligation method (Kamiya and Kuroda 1956) or by replacing the cell sap with artificial solutions of various ionic compositions and varied osmolarities using the vacuolar perfusion/cell ligation method (Tazawa 1964). In the former method the cell osmotic pressure was modified by dilution or concentration of the natural cell sap. In the latter method the artificial cell sap contained KCl, NaCl, CaCl_2_, and mannitol which was used to modify the cell osmotic pressure. These solutes are assumed to be impermeant, since the turgor pressure which is the difference between the osmotic pressure of the cell and that of the external medium remained constant. Kiyosawa and Tazawa (1972) found that the hydraulic resistance of the membrane (*L*p_m_^−1^) was not affected by wide variations in the concentrations of ions but strongly affected by varying the osmotic pressure of the vacuole. Analysis of the results showed that *L*p_m_^−1^ is related linearly to the external (π_o_) and the internal (π_i_) osmotic pressures in the following empirical equation (Eq. 1).

1 $${{L\mathrm{p}}_{\mathrm{m}}}^{-1}=0.265+0.045{\pi }_{\mathrm{i}}+0.022{\pi }_{\mathrm{o}}\left(\times {10}^{12}{\mathrm{m}}^{-1}\mathrm{s Pa}\right)$$

Here it is to be noted that the Eq. (1) was transformed from the original one presented by Kiyosawa and Tazawa (1972) in which the transcellular hydraulic resistance (2*L*p_m_^−1^) was used and the units of time and pressure were shown in min and atm, respectively.

Permeant solutes were also tested for their effects on *L*p in characean cells by Tazawa and Kamiya (1966). They reported that 5% (1.24 M) methanol decreased the *L*p of *Nitella* cells by 44% and 2% (0.34 M) ethanol decreased the *L*p by 24%. Kiyosawa (1975) also studied the effect of monohydric alcohols (methanol, ethanol, 1-propanol, 1-butanol, 1-pentanol) on the *L*p in *Nitella* cells and found that the hydraulic resistance of the cell (*L*p^−1^ in relative values) increased linearly with an increase in the concentration (C) of monohydric alcohols, and the slope of the *L*p^−1^-C curve became steeper with an increase of carbon chain length.

Likewise, Ye et al. (2004) found that in cells of *Chara corallina*, permeant glycol ethers, including ethylene glycol monomethyl ether (EGMME), diethylene glycol monomethyl ether (DEGMME), and triethylene glycol monoethyl ether (TEGMEE) also inhibited *L*p (increased *L*p^−1^) in a concentration-dependent manner. They also found that the larger the molecular weight of the solute, the greater was the inhibition.

The results obtained so far on the effect of impermeant and permeant solutes on the hydraulic conductivity (*L*p) or on the hydraulic resistance (*L*p^−1^) in characean cells inform that both types of solutes decrease *L*p (or increase *L*p^−1^), and the effect depends on the concentration of the external solution. However, in permeant solutes, the effect is dependent on the molecular size, which is not the case for non-permeant solutes.

Kiyosawa (1975) assumed that the permeant solutes decreased *L*p by narrowing the pore of the water channels. Alternatively, Ye et al (2004) proposed that the water channels were gated by the cohesion-tension (C/T) mechanism. According to this mechanism, the water molecules form a single file in the water channel and the solute molecules at the mouth of water channel exert a negative pressure inside the water channel, which causes a decrease in the hydraulic conductivity of the channel. Ye et al (2004) stressed that for permeant osmolytes, the molecular size is important in inhibiting or gating the water channels since the larger the solute molecules the stronger the negative tension evoked in the water channel. The cohesion-tension theory emphasizing the involvement of the molecular size of permeant solutes cannot account for the effect of impermeant solutes since their osmotic effect is independent of the molecular size.

We propose that it is not the molecular size of the solute but the effective osmotic pressure (^ef^π) that determines the hydraulic resistance. The effective osmotic pressure is related to the osmotic pressure by the following formula:

2 $${}^{\mathrm{ef}}\pi ={\sigma }_{\mathrm{s}}\pi$$

where σ_s_ is the reflection coefficient of the solute.

The object of the present study is to determine if the factor that controls the hydraulic conductivity by permeant solutes is the effective osmotic pressure or not. Since after permeating the membrane, the permeant solute affects *L*p_m_^−1^ from outside and inside of the plasma membrane, Eq. (1) can be used to assess the dependence of *L*p_m_^−1^ on the effective osmotic pressure.

By introducing the reflection coefficient (σ_s_), Eq. (1) is transformed into Eq. (3)

3 $${{L\mathrm{p}}_{\mathrm{m}}}^{-1}=0.265+0.045\left({\pi }_{\mathrm{c}}+{\upsigma }_{\mathrm{s}}{\pi }_{\mathrm{o}}\right)+0.022{\upsigma }_{\mathrm{s}}{\pi }_{\mathrm{o}}\left(\times {10}^{12}{\mathrm{m}}^{-1}\mathrm{s} \mathrm{Pa}\right)$$

where π_c_ is the osmotic pressure of the cell. The *L*p_m_^−1^ was estimated for the osmotic pressures (π_o_) of permeant solutes used in the experiment, and the relative values of *L*p_m_^−1^ (^r^*L*p_m_^−1^) were calculated. Then, both the observed and estimated values of ^r^*L*p_m_^−1^ were plotted against the concentration (C_s_) of all the permeant solutes, including the monohydric alcohols and the glycol ethers. The relation between C_s_ and ^r^*L*p_m_^−1^ was expressed in the following Eq. (4).

4 $${}^{\mathrm{r}}{{L\mathrm{p}}_{\mathrm{m}}}^{-1}={\uprho }_{\mathrm{m} }{C}_{\mathrm{s}}+1$$

where the hydraulic resistance modifier coefficient of the membrane for a specific solute (ρ_m_) was obtained on one hand directly from the observed values of ^r^*L*p_m_^−1^ and on the other hand from estimation of ρ_m_ by using the relationship between ρ_m_ and σ_s_ (Eq. 16) which was derived from modification of Eq. (3). The observed and estimated ρ_m_ values were plotted against the molecular weight (MW). The slope of the ρ_m_-MW curve and the correlation coefficients were compared between the observed and estimated ρ_m_ values. The fact that the estimated slope is close to the observed one suggests that the decisive factor of the solute controlling the hydraulic resistance of the water channel is not its molecular size but the effective osmotic pressure.

## Material and Methods

### Plant material

Throughout the experiments internodal cells of *Chara corallina* were used. The alga was cultured outdoors in buckets containing tap water. In winter each bucket was covered with a plate of polyacrylate resin and a sheet of polyethylene to avoid freezing. Internodal cells isolated from neighboring internodal cells were stored in tap water. Before each experiment cells were transferred to deionized water with an electrical conductivity of less than 10^–4^ S m^−1^) that was prepared by passing the tap water through a Cartridge Deionizer (Type G-10C, Organo, Tokyo).

### Test solutions

Sorbitol solutions were used to induce transcellular osmosis. The osmotic pressures of experimental solutions were measured with a WESCOR vapor pressure osmometer (MODEL 5520, WESCOR Inc., UT, U.S.A.).

### Measurement of cell osmotic pressure

The osmotic pressure of the cell was measured using the turgor balance method (Tazawa 1957).

### Determination of the hydraulic resistance of the cell (*L*p^−1^) by transcellular osmosis

**Fig. 1:**
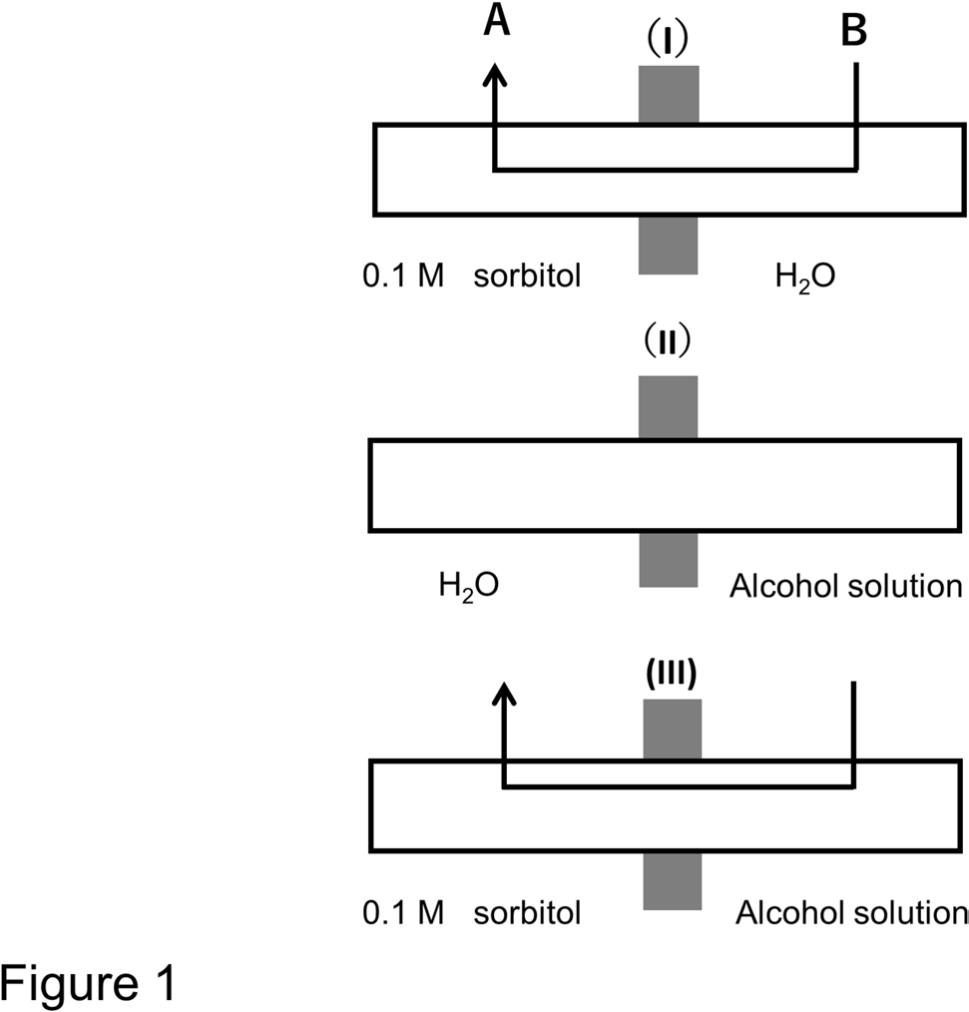
Measurement of the hydraulic resistance of a *Chara* cell by means of the transcellular osmosis before and after treatment of the half cell with an alcohol solution. The cell is partitioned into equal halves, one half in the chamber A and the other half in the chamber B. Both chambers are filled with water. (I) Transcellular osmosis is induced by replacing water in A with 0.1 M sorbitol. (II) Water in the chamber B is replaced with an alcohol solution. The alcohol enters the cell at B until its internal concentration reaches the external one. (III) Transcellular osmosis is induced by 0.1 M sorbitol under the condition that the half cell in B has been treated with an alcohol solution

The hydraulic resistance (*L*p^−1^) of an internodal cell was measured by the method of transcellular osmosis (Osterhout 1949; Kamiya and Tazawa 1956; Dainty and Ginzburg 1964a). Details of the measurement and the measuring apparatus were described in Tazawa et al. (2021). In brief, an internodal cell was placed in a double-chamber osmometer (A and B in Fig. 1) in such a manner to divide the cell into equal halves. First, both chambers were filled with deionized water. Transcellular osmosis was induced by replacing the water in the chamber A with 0.1 M sorbitol solution (Fig. 1-I). The volume of water transported from B to A transcellularly was indicated by a shift of the air babble placed in the capillary that was connected to the chamber B of the osmometer. *L*p was calculated from the volume of water moved in 60 s using a kinetic equation (Kamiya and Tazawa 1956; Tazawa et al. 2021).

### Determination of the hydraulic resistance of the cell wall (*L*p_w_^−1^)

The hydraulic resistance of the cell wall was measured in the cell wall tube prepared from an internodal cell. Details of the method are described in Kamiya et al. (1962) and Tazawa et al. (2021). Briefly, the cell wall tube was made by cutting one end of the cell placed in 0.3 M sorbitol solution in which the cell lost its turgor. The open end of the cell wall tube was tightly fitted to the tip of the measuring pipette with sticky wax. The other end of the pipette is connected to the pressure controlling and pressure measuring apparatus. The pressure used for driving water flow across the cell wall tube was 5 × 10^4^ Pa. The volume of water transported was indicated by the shift of an air bubble placed in the capillary of the pipette.

### Measurement of the effect of an alcohol on the hydraulic resistance (*L*p^−1^): half-cell treatment method

In the former studies to see the effect of an alcohol on *L*p (Tazawa and Kamiya 1965; Kiyosawa 1975), the whole cell was treated with the alcohol solution. The transcellular osmosis was induced by replacing the alcohol solution on one side (*A*) with the alcohol solution containing 0.2 M saccharose (Tazawa and Kamiya 1965) or 0.1 M mannitol (Kiyosawa 1975).

In the present study the half-cell was treated with an alcohol (half-cell treatment method). First, the control *L*p was determined by inducing transcellular osmosis with 0.1 M sorbitol (I in Fig. 1). After the measurement, water on the B-side was replaced with an alcohol solution, say 1.0 M methanol (II in Fig. 1). After 900 s, the transcellular water movement induced by methanol ceased, indicating that the internal concentration of methanol became equal to the external one. Subsequently transcellular osmosis was induced by replacing water on the A-side with 0.1 M sorbitol (III in Fig. 1) to determine the* L*p^−1^ of the cell whose half-cell had been treated with the alcohol solution.

The initial rate of transcellular osmosis (dv/dt)_i_ is proportional to the osmotic pressure (π_o_) of the sorbitol solution (Kamiya and Tazawa 1956). The proportionality constant or the transcellular osmotic constant (Kamiya and Tazawa 1956) is denoted as *K*. Referring to the control value of *K* obtained before treatment of the alcohol solution as *K*_1_ and *K* obtained after treatment of the cell half with the alcohol solution as *K*_2_, the ratio *K*_2_ / *K*_1_ is referred to β.

5 $$\upbeta ={K}_{2}/{K}_{1}$$

Let *L*p of the cell half on the B-side treated with an alcohol be denoted as ^alc^*L*p. Then the ratio (α) between ^alc^*L*p and the control *L*p is indicated by the next equation.

5-1 $${\alpha =}^{\mathrm{alc}}L\mathrm{p}/L\mathrm{p}$$

The relationship between α and β can be obtained in the following way. Let the surface area of the cell half be denoted by S. Then the transcellular osmotic resistance in the control osmosis (*K*_1_^−1^ in Fig. 1-I) is expressed in Eq. (5–2).

5-2 $${{K}_{1}}^{-1}=2\left({L\mathrm{p}}^{-1}/\mathrm{S}\right)$$

In the second transcellular osmosis where the cell half has been treated with an alcohol (Fig. 1-III), the transcellular osmotic resistance *K*_2_^−1^ is expressed in Eq. (5–3).

5-3 $${{K}_{2}}^{-1}={L\mathrm{p}}^{-1}/\mathrm{S}+{\alpha }^{-1}{L\mathrm{p}}^{-1}/\mathrm{S}={L\mathrm{p}}^{-1}/\mathrm{S}\left(1+{\alpha }^{-1}\right)$$

5-4 $$\upbeta ={K}_{2}/{K}_{1}={{K}_{1}}^{-1}/{{K}_{2}}^{-1}=2\alpha /\left(1+\alpha \right)$$

Then α is related to β in Eq. (6)

6 $${}^{\mathrm{alc}}L\mathrm{p}/L\mathrm{p}=\alpha =\upbeta /\left(2-\upbeta \right)$$

After measurement of ^alc^*L*p, the cell was rinsed with water and the cell wall tube was prepared. The *L*p_w_ of the cell wall tube immersed in water was measured. To see the effect of an alcohol on *L*p_w_ the cell wall tube was immersed in the alcohol solution and *L*p_w_ was measured.

The hydraulic resistance of the membrane *L*p_m_^−1^ was calculated from *L*p^−1^ and *L*p_w_^−1^ using Eq. (7).

7 $${L\mathrm{p}}^{-1}={{L\mathrm{p}}_{\mathrm{w}}}^{-1}+{{L\mathrm{p}}_{\mathrm{m}}}^{-1}$$

Since the hydraulic resistance of the tonoplast was shown to be much lower than that of the plasma membrane (Kiyosawa and Tazawa 1977; Tazawa et al. 2021), the hydraulic resistance of the plasma membrane is approximated by *L*p_m_^−1^.

The half-cell treatment has two advantages over the whole cell treatment. One is that the sorbitol concentration used to drive transcellular osmosis can be kept constant without interference of the solute, since the same sorbitol solution (0.1 M sorbitol) was used to induce the transcellular osmosis before and after treatment of the cell with the alcohol. The method has an additional advantage in that one is able to find promptly any anomalous osmosis caused by injury of the membrane that occurs in the cell half treated with alcohol. The anomalous osmosis was observed when the half-cell was treated with 1 M 1-propanol. Measurement of the hydraulic resistance was done after the cell half (B in Fig. 1) had been treated with 1 M 1-propanol for 900 s or more. The transcellular osmosis from B to A (Fig. 1-II) was induced with 0.1 M sorbitol. After 60 s 0.1 M sorbitol in A was replaced by water. The backward osmosis from A to B took place. Normally the backward osmosis ceased after 600 s. But in case where the cell half was treated with 1.0 M 1-propanol, often the backward osmosis did not cease and continued even after 600 s. In such a cell, the cytoplasmic streaming on the alcohol side of the cell (B in Fig. 1) was not observed, while that on the water side (A) was active. The occurrence of anomalous osmosis and inhibition of cytoplasmic streaming mean that long exposure of the cell to 1-propanol is toxic to the cell, causing loss of the semipermeable nature of the plasma membrane. The data from cells which showed the anomalous osmosis after 600 s of the backward osmosis were discarded.

Values of *L*p^−1^ and *L*p_m_^−1^ are shown in relative values of the control to correct the dispersion of the data caused by dispersion of the cell wall thickness (Tazawa et al. 2021).

### Estimation of the hydraulic resistance of the membrane (*L*p_m_^−1^) of Chara cells treated with glycol ethers

Ye et al. (2004) found that glycol ethers which are permeant to the plasma membrane inhibited the hydraulic conductivity of cells of *Chara corallina* in a concentration-dependent manner. The glycol ethers used were ethylene glycol monomethyl ether (EGMME), diethylene glycol monomethyl ether (DEGMME) and triethylene glycol monoethyl ether (TEGMEE), the molecular weights of which are 76, 120 and 178, respectively.

To estimate the values of *L*p_m_^−1^ of *Chara* cells treated with glycol ethers the average relative values of *L*p (^r^*L*p) which are presented in Fig. 4 of Ye et al. (2004) were used as the original data. First the values of ^r^*L*p^−1^ were calculated from the ^r^*L*p (Supplementary Fig. S2). Second, to estimate the values of *L*p^−1^, each value of ^r^*L*p^−1^ was multiplied by the average control value of *L*p^−1^ obtained in the present study which amounted to 0.67 ± 0.17 × 10^12^ m^−1^ s Pa (n = 24). Third, to estimate the values of *L*p_m_^−1^, the average value of* L*p_w_^−1^ obtained in the present study which amounted to 0.21 ± 0.17 × 10^12^ m^−1^ s Pa (n = 24) was subtracted from the value of *L*p^−1^. This value was applied to the *L*p_w_^−1^ under the assumption that *L*p_w_^−1^ was not changed by treatment of cells with glycol ethers. This assumption was supported by the following experiment. First the *L*p_w_ of a cell wall tube was measured in water. Then the cell wall tube was immersed in 0.5 M TEGMEE solution which induced the flow of water from the cell wall tube to the solution. The outflow of water stopped after 20 min. Measurement of *L*p_w_ in 0.5 M TEGMEE was conducted at a time of 60 min later. The relative values of *L*p_w_^−1^ of two cell wall tubes bathed in 0.5 M TEGMME were 1.00 and 0.97, respectively, showing that the *L*p_w_ was not affected by bathing the cell wall in the TEGMME medium.

### Determinaton of the reflection coefficient σ_s_ of the membrane for monohydric alcohols

Dainty and Ginzburg (1964b) determined the reflection coefficients of the membrane for permeant solutes in *Nitella translucens* and *Chara corallina*. They used two methods. One was to measure the initial rate of the transcellular osmosis induced by 0.1 or 0.2 M sucrose and by an equimolar permeant solute. Designating the initial rate of osmosis *R*, we get *R*_0_ from the initial (30 s) osmosis induced by the sucrose solution and *R*_s_ from the second osmosis induced by the same concentration of a permeant solute. σ_s_ can be calculated as *R*_s_/*R*_0_.

The second method is called the null method. First both chambers A and B (Fig. 1) were filled with 0.1 M sucrose solution. Then, the sucrose solution in A was replaced for a permeant solute solution with a concentration C_s_ and the rate of osmosis and its direction were registered. The experiment was repeated for three different values of C_s_. The value of Cs which caused no osmosis was determined by interpolation. The reflection coefficient of the membrane for a solute (σ_s_) was obtained as σ_s_ = 0.1 M/C_s_. The null method may be referred to the osmotic equilibrium method.

In the present study, transcellular osmosis was induced by 0.1 M sorbitol in A as shown in Fig. 1-I The volume of water moved at 5 s was registered as v^5^. Then, both cell ends were immersed in 0.1 M sorbitol. For instance, the sorbitol solution in A was changed to 0.25 M ethanol. When the effective osmotic pressure of 0.25 M ethanol is equal to that of 0.1 M sorbitol, no initial water flow would be observed. Then σ_s_ is calculated as 0.1/0.25 = 0.4. In case when a small water flow from B to A at 5 s amounting to *v*_s_^5^ was observed, this volume of water flow was converted to the concentration of sorbitol (Δ*C*) by the following equation: Δ*C* = (v_s_^5^ / v^5^) × 0.1 M (sorbitol). The sorbitol concentration which is iso-osmotic to 0.25 M ethanol is calculated to be (0.1 + Δ*C*) M. The σ_s_ was calculated as (0.1 + Δ*C*)/0.25. When the water flow was from A to B, σ_s_ was calculated as (0.1-Δ*C*)/0.25.

### Statistics

Student’s *t*-tests were conducted to test the significant difference between the relative values of hydraulic resistance of the membrane (^r^*L*p_m_^−1^) and σ_s_ values obtained from different alcohols in Fig. 3 and Table 1, respectively. Significant differences between alcohols (p < 0.05) are marked with an asterisk.

**Table 1:** Reflection coefficients (${\sigma }_{\mathrm{s}}$) and molecular weight (MW) of permeant solutes

Solute	MW	${\sigma }_{\mathrm{s}}$(20°C)	${\sigma }_{\mathrm{s}}$(25°C)	
Methanol	32	0.34 ± 0.05	0.28 ± 0.04	This study
Ethanol	46	0.47 ± 0.06	0.37 ± 0.05	This study
1-Propanaol	60	0.42 ± 0.05	0.33 ± 0.05	This study
EGMME	76		0.59 ± 0.03	Ye et al. (2004)
DEGMME	120		0.78 ± 0.05	Ye et al. (2004)
TEGMEE	178		0.82 ± 0.07	Ye et al. (2004)

Values of σ_s_ at 20⁰C were measured by the osmotic equilibrium method but those at 25⁰C were estimated by using the data of Herzel and Steudle (1997) who found that theσs of ethanol and 1-propanol were at 30⁰C were by 33% and 41% less than those at 20⁰C, respectively. Values at 25⁰C were approximated to be 20% less than those at 20⁰C

## Results

### A. Monohydric alcohols

#### A-1. Hydraulic resistances of the cell (*L*p^−1^), the cell wall (*L*p_w_^−1^) and the membrane (*L*p_m_^−1^) affected by methanol and ethanol: an example

**Fig. 2:**
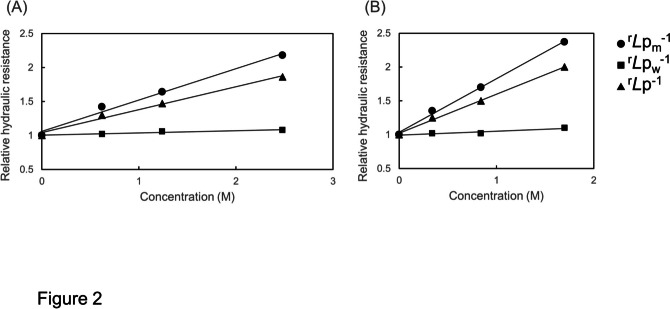
An example of relative hydraulic resistances of the cell (^r^*L*p^−1^: triangles), the cell wall (^r^*L*p_w_^−1^: squares) and the membrane (^r^*L*p_m_^−1^: circles) in relation to the concentration of methanol (A) and ethanol (B) in a cell of *Chara corallina* (sample: #397, cell 1). The slopes of regression lines for ^r^*L*p^−1^, ^r^*L*p_w_^−1^ and ^r^*L*p_m_^−1^ in methanol are 0.337, 0.033 and 0.461 M^−1^, respectively, and those in ethanol are 0.577, 0.057 and 0.79 M^−1^, respectively. The correlation coefficients (R) for ^r^*L*p^−1^, ^r^*L*p_w_^−1^ and ^r^*L*p_m_^−1^ are in methanol 0.994, 0.962 and 0.993, respectively, and those in ethanol are 0.998, 0.944 and 0.998, respectively

Figure 2 shows the hydraulic resistances of the cell (*L*p^−1^), the cell wall (*L*p_w_^−1^) and the membrane (*L*p_m_^−1^) in relative values (^r^*L*p^−1^, ^r^*L*p_w_^−1^, ^r^*L*p_m_^−1^) in relation to the concentration of methanol (A) and ethanol (B). The data of *L*p^−1^ and *L*p_w_^−1^ were obtained from cell #397 and shown in supplementary Fig. S1 with *L*p_m_^−1^ which were calculated by Eq. (7).

In Fig. 2 the slope of the curve is named as the hydraulic resistance modifier coefficient and marked as ρ. ρ is specified for the cell as ρ_c_, for the cell wall as ρ_w_ and for the membrane as ρ_m_. For methanol and ethanol, the ρ_c_ is 0.34 and 0.58 M^−1^, respectively, the ρ_m_ is 0.46 and 0.79 M^−1^, respectively and the ρ_w_ is 0.033 and 0.057 M^−1^, respectively. The very low value of ρ_w_ suggests that the *L*p_w_^−1^ is almost independent of the concentration of the alcohols.

#### A-2. Relative hydraulic resistances of the cell (^r^*L*p^−1^), the cell wall (^r^*L*p_w_^−1^) and the membrane (^r^*L*p_m_^−1^) versus the concentration of monohydric alcohols: collective data

The experiment to see the effects of monohydric alcohols on the hydraulic resistances of the cell (*L*p^−1^) and the cell wall (*L*p_w_^−1^) shown in A-1 was carried out also in other cells. The alcohols tested were methanol, ethanol and 1-propanol. Values of *L*p_m_^−1^ were calculated by subtracting *L*p_w_^−1^ from *L*p^−1^. *L*p_w_^−1^ was shown to be hardly affected by the alcohols in the concentration range tested (Supplementary Table S2).

**Fig. 3:**
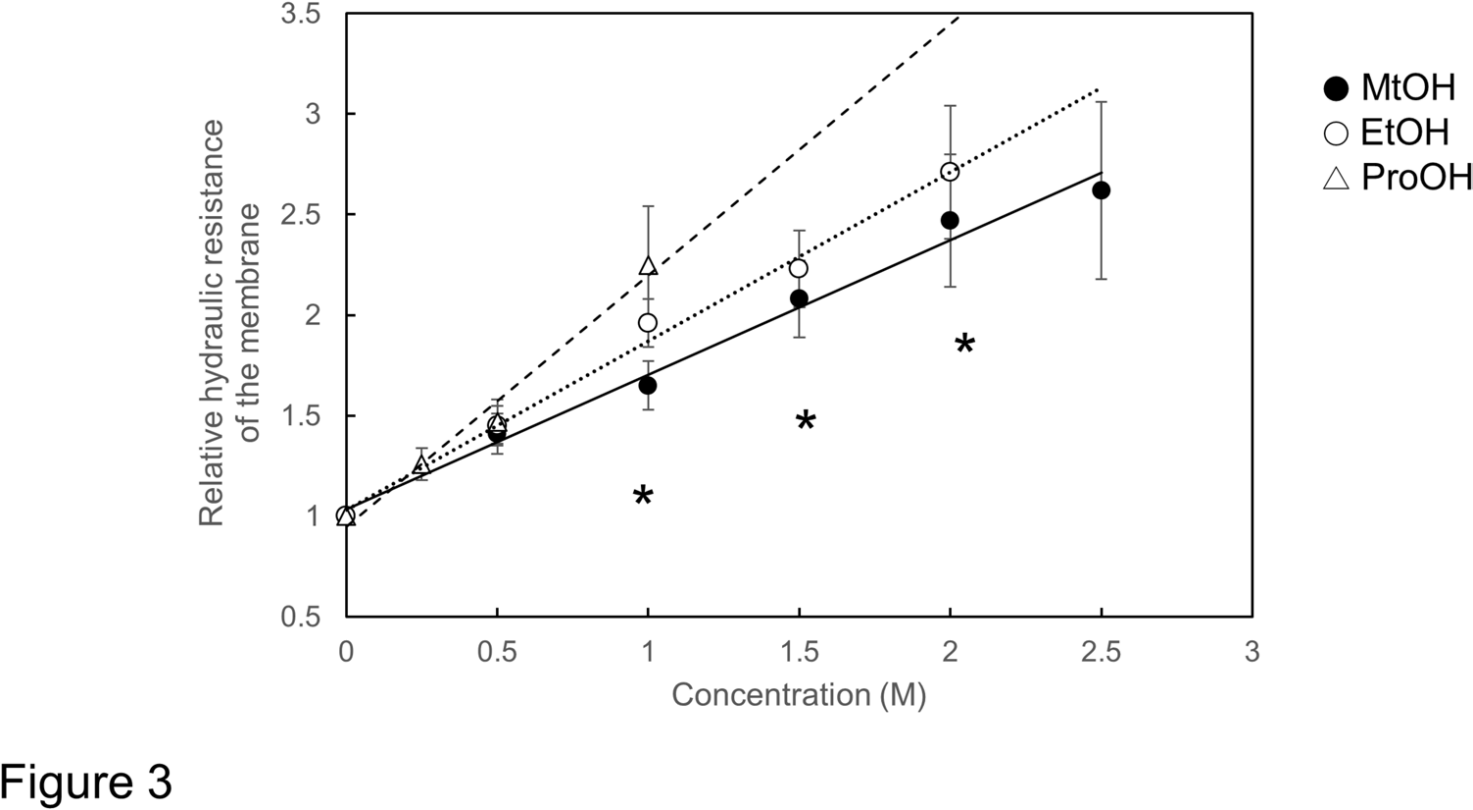
Relative hydraulic resistance of the membrane versus concentration (in M) of methanol (closed circles) up to 2.5 M, ethanol (open circles) up to 2 M and 1-propanol (open triangles) up to 1 M in cells of *Chara corallina*. Data are the means ± SD (n = 5 to 18). Significant differences are indicated by asterisks (Student’s *t*-test, *p* < 0.05) between methanol, ethanol and/or 1-propanol in each concentration. The slopes of approximation lines for methanol, ethanol and 1-propanol are 0.62, 0.84 and 1.25 M^−1^, respectively. The correlation coefficients (R) for methanol, ethanol and 1-propanol are 0.998, 0.996 and 0.991, respectively

Figure 3 shows the collective data of the relative hydraulic resistances of the membrane (^r^*L*p_m_^−1^) in relation to the concentrations of methanol (●), ethanol (○) and 1-propanol (△). In all alcohols the ^r^*L*p_m_^−1^ increased linearly with the increase in their concentration. The correlation coefficients (R) were 0.998 for methanol, 0.996 for ethanol and 0.991 for 1-propanol, showing a high correlation between the ^r^*L*p_m_^−1^ and the concentration in all alcohols tested. The slope of the curve (ρ_m_) was 0.62 M^−1^ for methanol, 0.84 M^−1^ for ethanol and 1.25 M^−1^ for 1-propanol showing that the effect of increasing the hydraulic resistance of the membrane increases with the increase in the number of carbons in the aliphatic carbon chain.

Figure 3 shows that the difference of ^r^*L*p_m_^−1^ values between methanol and ethanol is significant (*p*-value < 0.05) at the concentrations of 1.0, 1.5 and 2.0 M, respectively, and that between ethanol and 1-propanol is significant at the concentration of 1.0 M.

The highest concentration of 1-propanol was 1 M because of its toxic effect at higher concentrations. Treatment of the half cell (B side in Fig. 1) for more than 900 s with 1 M 1-propanol often resulted in a loss of the semipermeable nature of the plasma membrane. The loss of semipermeability of the membrane was visualized by the occurrence of anomalous transcellular osmosis without a transcellular driving force, namely when both compartments A and B were filled with water.

### B. Estimation of the hydraulic resistance of the membrane (^r^*L*p_m_^−1^) affected by glycol ethers using the data of Ye et al. (2004)

Ye et al. (2004) found that glycol ethers which are permeant to the plasma membrane inhibited the hydraulic conductivity of cells of *Chara corallina* in a concentration-dependent manner. The glycol ethers used were ethylene glycol monomethyl ether (EGMME), diethylene glycol monomethyl ether (DEGMME) and triethylene glycol monoethyl ether (TEGMEE), the molecular weights of which are 76, 120 and 178, respectively.

Figure 4 in Ye et al. (2004) shows the relative *L*p values (^r^*L*p) of cells treated with 0.2, 0.4, 0.6 and 0.8 M ethylene glycols. From these values the corresponding ^r^*L*p^−1^ values were calculated. To know the ^r^*L*p_m_ it is necessary to know the *L*p_m_^−1^. The *L*p_m_^−1^ can be calculated if we know the values of *L*p^−1^ and *L*p_w_^−1^. Values of *L*p^−1^ and *L*p_w_^−1^ were estimated by using the average control values of *L*p^−1^ and *L*p_w_^−1^ (*L*p^−1^ and *L*p_w_^−1^ obtained in water) as explained in Material and Methods.

**Fig. 4:**
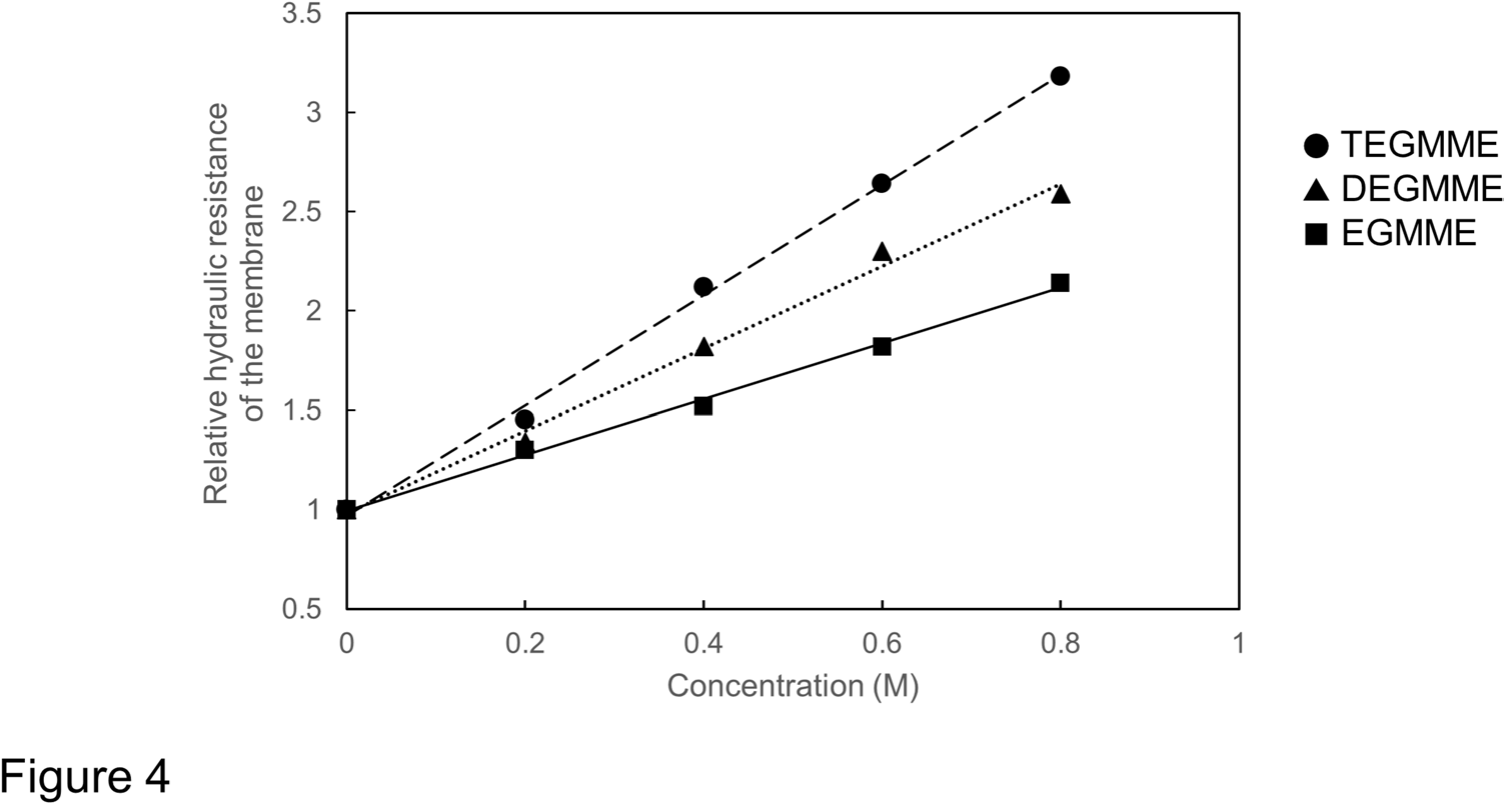
Relative hydraulic resistance of the membrane (^r^*L*p_m_^−1^) in relation to the concentration (in M) of glycol ethers, EGMME (closed squares), DEGMME (closed triangles) and TEGMME (close circles). The slopes of the regression lines for EGMME, DEGMME and TEGMME are 1.4, 2.07 and 2.78 M^−1^, respectively. The correlation coefficients (R) for EGMME, DEGMME and TEGMME are 0.998, 0.997 and 0.997, respectively. See the text for calculation of ^r^*L*p_m_^−1^ of glycol ethers

Calculated values of *L*p_m_^-1^ were converted to relative values (^r^*L*p_m_^-1^), which are shown in Fig. 4 in relation to the concentration of glycol ethers. The ^r^*L*p_m_^-1^ increases linearly with an increase in the concentration in all glycol ethers. The slopes of the curves (ρ_m_) for EGMME, DEGMME and TEGMEE are 1.4, 2.07 and 2.78 M^-1^, respectively. The respective correlation coefficients (R) amounting to 0.998, 0.996 and 0.998 show that the hydraulic resistance of the membrane is highly correlated to the concentration of three species of glycol ethers.

### C. Membrane hydraulic resistance modifier coefficients (ρ_m_) versus molecular weight of permeant solutes

**Fig. 5:**
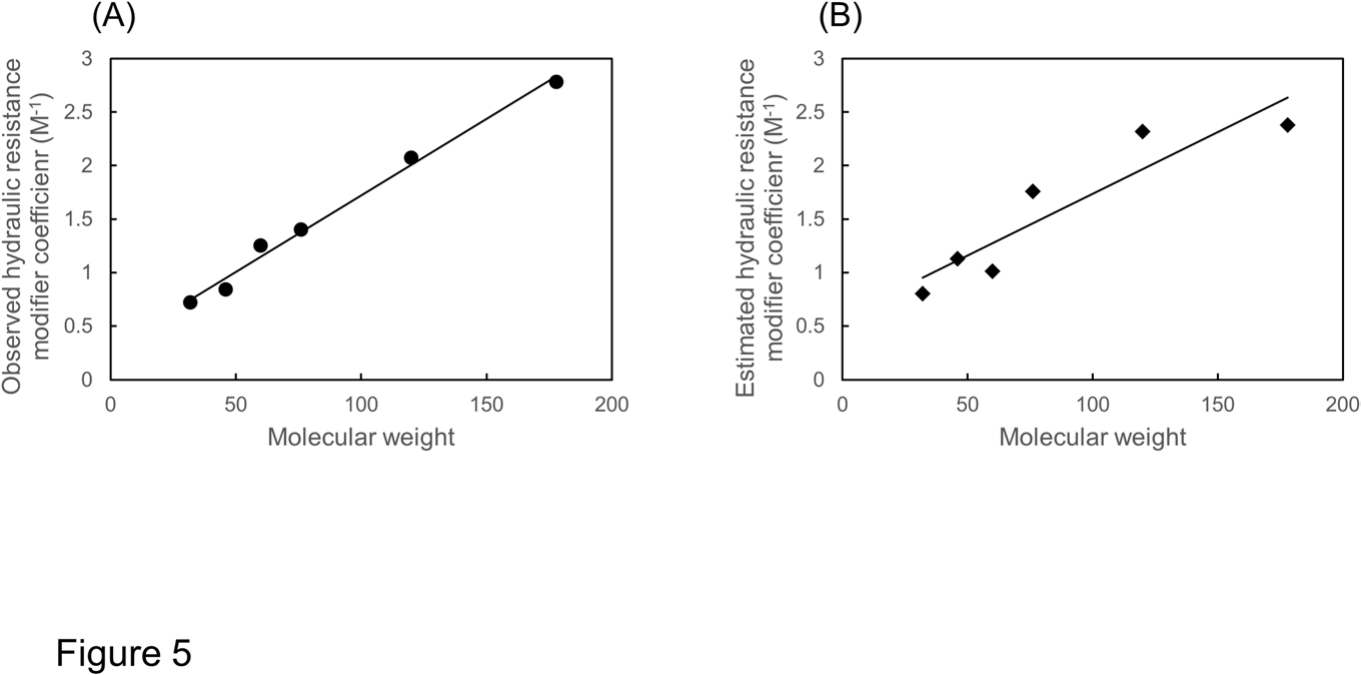
Observed **(A,** ρ_m_ in M^−1^) and estimated (B, ^est^ρ_m_ in M^−1^) hydraulic resistance modifier coefficients of the membrane in relation to the molecular weights of permeating osmolytes including methanol, ethanol, 1-propanol, EGMME, DEGMME and TEGMEE. The slopes of the regression lines for ρ_m_ (A) and ^est^ρ_m_ (B) are 0.014 and 0.012, respectively. The correlation coefficients (R) for ρ_m_ and ^est^ρ_m_ are 0.995 and 0.917, respectively

In Fig. 5A the membrane hydraulic resistance modifier coefficients (ρ_m_) of three monohydric alcohols and three glycol ethers are plotted against the molecular weights (32, 46, 60, 76, 120 and 178). The regression line with the correlation coefficient (R) of 0.995 shows that ρ_m_ is highly correlated to the molecular weight.

#### D. Determination of reflection coefficients (σ_s_) of monohydric alcohols

Dainty and Ginzburg (1964a, b) were of the opinion that the value of σ_s_ determined by the null method is more accurate than the value measured by the initial rate method. In the latter method the osmotic driving force of a rapidly permeating osmolyte is decreased quickly within the measuring time (30 s) by permeation of the osmolyte. This causes a lower value of σ_s_ than the null method. For instance the σ_s_ of ethanol obtained by the initial rate method was 0.29, while that obtained by the null method was 0.4 (p.134 in Dainty and Ginzburg 1964b). In the present study σ_s_ was obtained by the osmotic equilibrium method which is practically the same as the null method. Table 1 shows the average σ_s_ values (0.34 for methanol, 0.47 for ethanol and 0.42 for 1-propanol).

Dainty and Ginzburg (1964b) found that in *Nitella translucens* σ_s_ of ethanol was strongly affected by the temperature. The σ_s_ of ethanol measured at 3, 15 and 25⁰C was 0.57, 0.43 and 0.23, respectively. Hertel and Steudle (1997) reported that in *Chara corallina* σ_s_ of ethanol and 1-propanol at 20⁰C were 0.36 and 0.22, respectively, and at 30⁰C were 0.20 and 0.13, respectively. The σ_s_ at 25⁰C for ethanol and 1-propanol in *Chara* cells are estimated to be 0.28 and 0.175, respectively. Then, the ratio ^25^σ_s_/ ^20^σ_s_ is calculated as 0.78 for ethanol and as 0.8 for 1-propanol. In the present study the temperature where σ_s_ determination for alcohols was carried out was about 20⁰C, while the temperature where *L*p^−1^ was measured was about 25⁰C. The σ_s_ measured at 20⁰C (^20^σ_s_) was corrected for the σ_s_ at 25⁰C (^25^σ_s_) by multiplying σ_s_^20^ with a factor 0.8. The estimated values of ^25^σ_s_ for methanol, ethanol and 1-propanol were 0.28, 0.37 and 0.33, respectively (Table 1). Since theσ_s_ values for glycol ethers were measured at 23–25 C (Ye et al. 2004), no correction for the temperature was made (Table 1).

Steudle and Tyerman (1983) found that the reflection coefficient (σ_s_) of ethanol decreased significantly with increasing concentration (Fig. 5 in Steudle and Tyerman (1983)). Later Ye et al. (2004) found that in permeant three glycol ethers σ_s_ tended to decrease with increasing concentration but the tendency in the changes is not statistically significant (Fig. 6 in Ye et al. (2004)). In the present study we found that the ^r^*L*p_m_^−1^ increased linearly with increasing concentration of monohydric alcohols (Fig. 3). The slope of the regression line (ρ_m_) is related to σ_s_ as shown in Eq. (15). This is inconsistentwith the previously observed decrease in the reflection coefficients with concentration.

#### Analysis of the data

In contrast to impermeant solutes, permeant solutes given to the outside of the cell exert their osmotic effects on the plasma membrane from both sides of the membrane. Kiyosawa and Tazawa (1972) studied the effects of extracellular (π_o_) and intracellular (π_i_) osmotic pressures on the hydraulic resistance (*L*p^−1^) in cells of *Nitella flexilis*. They found that *L*p^−1^ was linearly dependent on both π_o_ and π_i_. After measurement of the hydraulic resistance of the cell wall (*L*p_w_^−1^) the hydraulic resistance of the membrane (*L*p_m_^−1^) was calculated. They formulated the experimental data into Eq. (8).

8 $${{L\mathrm{p}}_{\mathrm{m}}}^{-1}=0.265+0.045{\pi }_{\mathrm{i}}+0.022{\pi }_{\mathrm{o}}\left(\times {10}^{12}{\mathrm{m}}^{-1}\mathrm{s Pa}\right)$$

It is to be noted that the Eq. (8) was based on the data obtained in cells of *Nitella flexilis.* Assuming that *Chara* cells respond to the extracellular and intracellular osmotic pressures similarly to *Nitella* cells, Eq. (8) was applied to the analysis of the data obtained in *Chara* cells.

As shown in Fig. 3 and in Fig. 4, the ^r^*L*p_m_^−1^ increased linearly with an increase in the concentration (C) of the permeant osmolytes with high correlation coefficients (R: methanol 0.998, ethanol 0.996, 1-propanol 0.990, EGMME 0.998, DEGMME 0.997, TEGMEE 0.999). The proportionality coefficient of the ^r^*L*p_m_^−1^-C curve, which is referred to the hydraulic resistance modifier coefficient of the membrane (ρ_m_), was used as the parameter showing the effectiveness of an osmolyte on increasing the membrane hydraulic resistance. The ρ_m_ values were obtained for monohydric alcohols (Fig. 3) and glycol ethers (Fig. 4). Plotting ρ_m_ values of these permeant osmolytes against their molecular weights, a linear relationship was obtained with a high correlation coefficient (R = 0.995 in Fig. 5A). The relationship was analyzed on the basis of the empirical formula (Eq. 8) under consideration of the reflection coefficients (σ_s_) of the permeant solutes.

Differing from non-permeant solutes the osmotic pressure of a permeant solute across the membrane is lower than that of an equimolar impermeant solute. After Dainty and Ginzburg (1964b), the reflection coefficient of a solute (σ_s_) is defined as the measure of selectivity of the membrane towards a given solute and is defined as

9 $${\sigma }_{\mathrm{s}}={\pi }_{\mathrm{s}}/{\mathrm{RTC}}_{\mathrm{s}}$$

where RTC_s_ is the theoretical osmotic pressure of a medium with the molar concentration of C_s_ and π_s_ is the effective osmotic pressure of the permeant solute solution.

Introducing the effective osmotic pressure of a permeant solute (s) into Eq. (8), *L*p_m_^−^.^1^ is transformed to Eq. (10)

10 $$\begin{array}{c}{{L\mathrm{p}}_{\mathrm{m}}}^{-1}=0.265+0.045\left({\pi }_{\mathrm{c}}+{\upsigma }_{\mathrm{s}}{\mathrm{RTC}}_{\mathrm{s}}\right)+{0.022}_{{\upsigma }_{\mathrm{s}}}{\mathrm{RTC}}_{\mathrm{s}}\\ =0.265+0.04{\pi }_{\mathrm{c}}+{0.067}_{{\upsigma }_{\mathrm{s}}}{\mathrm{RTC}}_{\mathrm{s}}\left(\times {10}^{12}{\mathrm{m}}^{-1} \mathrm{s} \mathrm{Pa}\right)\end{array}$$

where π_c_ is the osmotic pressure of the cell. Since the average cell osmotic concentration was 0.27 M sorbitol equivalent, π_c_ is calculated to be 6.69 × 10^5^ Pa at 298 K. RTC_s_ is the theoretical osmotic pressure of an osmolyte solution. Introducing 6.69 × 10^5^ Pa for π_c_, Eq. (10) is transformed to Eq. (11).

11 $${{L\mathrm{p}}_{\mathrm{m}}}^{-1}=0.57+0.067{\upsigma }_{\mathrm{s}}{\pi }_{\mathrm{s}}\left(\times {10}^{12}{\mathrm{m}}^{-1}\mathrm{s Pa}\right)$$

The relative value of the hydraulic resistance of the membrane (^r^*L*p_m_^−1^) is expressed in Eq. (12).

12 $${}^{\mathrm{r}}{{L\mathrm{p}}_{\mathrm{m}}}^{-1}={{L\mathrm{p}}_{\mathrm{m}}}^{-1}/0.57=1+0.12{\sigma }_{s}{\pi }_{s}$$

or in Eqn. (13).

13 $${}^{\mathrm{r}}{{L\mathrm{p}}_{\mathrm{m}}}^{-1}=1+0.12{\sigma }_{\mathrm{s}}{\mathrm{RTC}}_{\mathrm{s}}$$

Regression lines showing the relation of ^r^*L*p_m_^-1^ to the concentration of either the monohydric alcohols (Fig. 3) or the glycol ethers (Fig. 4) are expressed in the general formula of Eqn. (14).

14 $${}^{\mathrm{r}}{{L\mathrm{p}}_{\mathrm{m}}}^{-1}=1+{\uprho }_{\mathrm{m}}{\mathrm{C}}_{\mathrm{s}}$$

Comparing Eq. (14) with Eq. (13), ρ_m_ is related to σ_s_ at T = 298 K in the following way:

15 $${\uprho }_{\mathrm{m}}=0.12{\sigma }_{\mathrm{s}}\mathrm{RT}=0.12\times 24.8{\sigma }_{\mathrm{s}}\left({10}^{-7} {\mathrm{Pa}}^{-1}\right)$$

or

16 $${\uprho }_{\mathrm{m}}=2.98{\sigma }_{\mathrm{s}}\left({\mathrm{M}}^{-1}\right)$$

Here the values of ρ_m_, calculated by introducing values of σ_s_ into Eq. (16), are referred to as the estimated ρ_m_ (^est^ρ_m_). Observed values of ρ_m_ for methanol, ethanol, 1-propanol, EGMME, DEGMME and TEGMEEG were 0.67, 0.84, 1.25 (Fig. 3), 1.4, 2.07 and 2.78 M^−1^ (Fig. 4), respectively. These values were plotted against the molecular weights of the permeant solutes to give Fig. 5A.

Values of ^est^ρ_m_ were calculated by introducing values of σ_s_ obtained at 25 °C (Table 1) into Eq. (16). The σ_s_ for methanol, ethanol and 1-propanol were measured at 20⁰C. Since the hydraulic resistance was measured at 25⁰C, the σ_s_ corrected for the temperature (Table 1) were applied to Eq. (16). Values of ^est^ρ_m_ calculated by Eq. (16) are 1.01, 1.4 and 1.25 for methanol, ethanol and 1-propanol, respectively, and 1.76, 2.32 and 2.38 for EGMME, DEGMME and TEGMEE, respectively.

Figure 5B shows the relationship between the values of ^est^ρ_m_ and the molecular weights (MW) of six permeant osmolytes. The slope of the curve is 0.012 M^−1^/MW which is close to the slope of the curve of observed ρ_m_ versus MW (0.014 M^−1^/MW, Fig. 5A).

## Discussion

The present study revealed that permeant monohydric alcohols increased the hydraulic resistance of the membrane (^r^*L*p_m_^−1^) in cells of *Chara corallina* linearly with an increase in the concentration of alcohols (Fig. 3). The alcohols used were methanol, ethanol and 1-propanol whose molecular weights are 32, 46 and 60, respectively. Similar experiments were carried out by Ye et al. (2004) using permeant glycol ethers in cells of *Chara corallina*. The glycol ethers used were ethylene glycol monomethyl ether (EGMME), diethylene glycol monomethyl ether (DEGMME), and triethylene glycol monoethyl ether (TEGMEE) with the molecular weights of 76, 120 and178. They found that the glycol ethers inhibited *L*p in a concentration-dependent manner. Using their data we calculated the values of *L*p_m_^−1^. As in monohydric alcohols the relative values of *L*p_m_^−1^ (^r^*L*p_m_^−1^) increased linearly with an increase in the concentration in all glycol ethers tested (Fig. 4). The results are in accord with the results obtained in *Nitella flexilis* by Kiyosawa (1975) although his data (Fig. 2) were not tested statistically.

The concentration-dependent behavior of ^r^*L*p_m_^−1^ of permeant osmolytes including monohydric alcohols and glycol ethers was analyzed on the basis of the effects of the intracellular and extracellular osmotic pressures on the hydraulic resistance of the membrane of *Nitella* cells found by Kiyosawa and Tazawa (1972).

As for the pathway(s) of osmotic water flow Henzler and Steudle (1995) proposed the composite membrane model. According to the model, the membrane is composed of two distinct arrays, proteinaceous arrays with specific water channels and lipid bilayer arrays. The former is mercury-sensitive and the latter may be mercury-insensitive. In cells of *Chara corallina* a water channel inhibitor HgCl_2_ at 0.05 mM inhibited *L*p by 90% (Schütz and Tyerman 1997) and at 1 mM by 96% (Tazawa et al. 1996). Consequently in *Chara* cells most of the osmotic water flow is assumed to occur via Hg-sensitive water channels. The increase in the hydraulic resistance caused by permeant solutes may be accounted for by an increase in the resistance of water movement through water channels. A similar interpretation was done by Kiyosawa (1975) who found that in *Nitella flexilis* monohydric alcohols (methanol, ethanol, n-propanol, n-butanol, n-pentano) increased the cell hydraulic resistance with increasing concentrations. He assumed that “alcohol molecules interact with the membrane to make the equivalent pore radius of the membrane narrower without changing the nature of the water flow”.

Present results show that the potential of permeant solutes to increase the hydraulic resistance of the plasma membrane is positively correlated with the molecular weight which is an index of the molecular size (Fig. 5A). Ye et al. (2004) proposed the cohesion/tension mechanism for the inhibition of hydraulic conductivity of water channels by osmolytes. Permeating solute molecules at the mouth of water channels exert a tension (negative pressure) within the water channel and would increase the hydraulic resistance of water channels. Ye et al (2004) in their Fig. 4 which depicts the cohesion/tension model explain the mechanism as follows:”Since solutes were excluded from aquaporins, tensions were set up in the pores which caused a reversible mechanical deformation of the protein as tensions (negative pressure) increased.” Here they assumed that “the larger the size of a solute, the higher the efficiency in exerting tensions within pores”. The linear relationship between the hydraulic resistance modifier coefficient and the molecular weight (Fig. 5A) seems to support this assumption.

However, impermeant solutes affect the hydraulic resistance independently of the species of the solutes including electrolytes and nonelectrolytes of varied molecular weights. Taking advantage of the vacuolar perfusion technique Kiyosawa and Tazawa (1972) varied the ionic composition and the osmotic pressure of the vacuolar sap by perfusing the vacuole with artificial solutions. They verified that the active factor affecting the hydraulic resistance is not ions but the osmotic pressure of the perfusion media. Impermeant solutes may exert the same magnitude of tension (negative pressure) within the water channel, irrespectively of their molecular sizes and ion species. In impermeant solutes σ_s_ is unity irrespective of the molecular weight (cf. Table 2 in Tyerman and Steudle 1982) and their values of ρ_m_ are assumed to be constant (2.98 M^-1^ from Eqn. 16).

In conclusion, the mechanism of permeant solutes to increase the hydraulic resistance is essentially the same as that of impermeant solutes in that the effective osmotic pressure of the solution is decisive in determining the water permeability of water channels.

## Supplementary Information

Below is the link to the electronic supplementary material.

Supplementary file1 (PDF 370 KB)

## Data Availability

N/A.

## Code availability

N/A.
