# Supplementary material for: Analysis of the effect of permeant solutes on the hydraulic resistance of the plasma membrane in cells of Chara corallina
Source: Protoplasma. 2024 Oct 23;262(2):385–95. doi: 10.1007/s00709-024-02000-6 (PMC11839782; doi:10.1007/s00709-024-02000-6)
Supplement: Supplementary file 1 — Supplementary file1 (PDF 370 KB) [file 709_2024_2000_MOESM1_ESM.pdf]

Analysis of the effect of permeant solutes on the hydraulic resistance of the plasma membrane in cells of *Chara corallina.*

Tazawa M., Wayne R., Katsuhara M.

Supplementary Figures and Tables

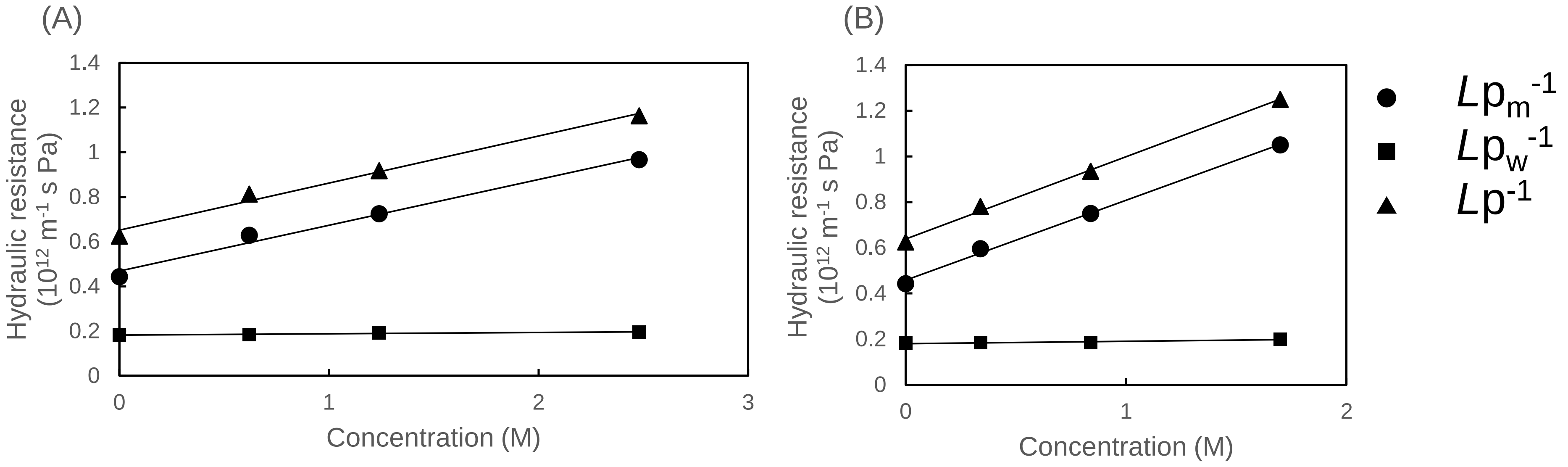

Supplementary Figure S1.
An example of hydraulic resistances (10^{12} m^{-1} s Pa) of the cell (Lp^{-1}: triangles), the cell wall (Lp_w^{-1}: squares) and the membrane (Lp_m^{-1}: circles) versus the concentration (in M) of methanol (A) and ethanol (B) in a cell of *Chara corallina* (sample: #397, cell 1). The concentration of methanol (v/v %) was 2.5, 5.0 and 10 % (0.62, 1.24, 2.48 M) and the concentration of ethanol was 2.0, 5.0 and 10 % (0.34, 0.86, 1.71 M).

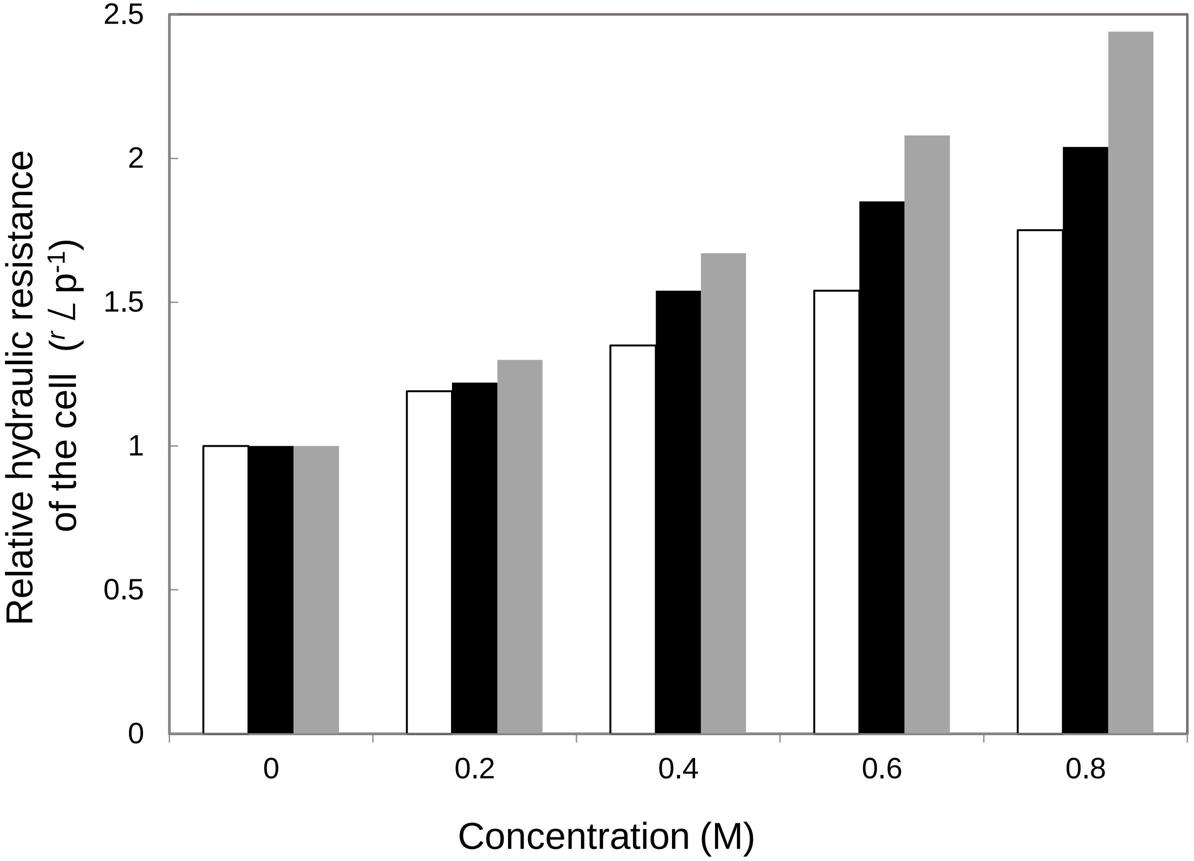

Supplementary Figure S2

Relative hydraulic resistance of the cell ($^r Lp^{-1}$) versus the concentration (in M) of glycol ethers, EGMME (white), DEGMME (black) and TEGMEE (grey). Values of $^r Lp^{-1}$ were calculated from the relative values of *L*p shown in Fig. 4 in Ye et al. (2004).

Supplementary Table S1

Hydraulic resistances of the cell (Lp^{-1}) and the membrane (Lp_m^{-1}) of *Chara collarina* in 10^{12} m^{-1} s Pa (Means and SD)

methanol	0	0.5	1	1.5	2	2.5 M
Lp^{-1}	0.64	0.73	1.02	0.95	1.33	1.18
SD	0.17	0.1	0.35	0.12	0.43	0.16
Lp_m^{-1}	0.41	0.54	0.74	0.79	1.09	0.93
SD	0.1	0.09	0.26	0.13	0.31	0.13
n	15	8	6	8	6	8

etthanol	0	0.5	1	1.5	2 M
Lp^{-1}	0.74	0.87	1.18	1.18	1.57
SD	0.21	0.17	0.31	0.2	0.12
Lp_m^{-1}	0.51	0.68	0.98	0.98	1.36
SD	0.13	0.16	0.22	0.19	0.29
n	18	12	13	10	7

1-propanol	0	0.25	0.5	1 M
Lp^{-1}	0.67	0.71	0.77	0.92
SD	0.1	0.15	0.11	0.18
Lp_m^{-1}	0.34	0.45	0.49	0.66
SD	0.1	0.16	0.2	0.17
n	10	5	10	6

Hydraulic resistance of the cell (Lp^{-1}) and the membrane (Lp_m^{-1}) treated with methanol (up to 2.5 M), ethanol (up to 2M) and 1-propanol (up to 1 M).

Supplementary Table S2

Relative hydraulic resistance of the cell wall ($^{r}Lp_{w}^{-1}$) of *Chara corallina* affected by alcohols (Mean±SD)				
Conc.	0 M	0.5 M	1 M	2 M
methanol	1	1.01±0.06	1.03±0.06	1.07±0.01
n	7	2	5	4
ethanol	1	0.99±0.05	1±0.04	1.06±0.06
n	8	4	3	6
1−propanol	1	0.99±0.02	1	
n	3	3	1	

Relative hydraulic resistance of the cell wall ($^{r}Lp_{w}^{-1}$) treated with methanol (up to 2 M), ethanol (up to 2 M) and 1-propanol (up to 1 M).
